# A Nutraceutical Approach to Menopausal Complaints

**DOI:** 10.3390/medicina55090544

**Published:** 2019-08-28

**Authors:** Pasquale De Franciscis, Nicola Colacurci, Gaetano Riemma, Anna Conte, Erika Pittana, Maurizio Guida, Antonio Schiattarella

**Affiliations:** 1 Department of Women, Child and General and Specialized Surgery, University of Campania “Luigi Vanvitelli”, 80138 Naples, Italy; 2 Department of Neuroscience, Reproductive Sciences and Dentistry, School of Medicine, University of Naples “Federico II”, 80138 Naples, Italy

**Keywords:** nutraceuticals, menopausal symptoms, natural approach, supplementation, menopause, isoflavones, *Agnus castus*, vasomotor symptoms, sleep disturbances

## Abstract

The menopausal transition, or perimenopause, is characterized by menstrual irregularities, vasomotor symptoms, sleep disturbances, mood symptoms, and urogenital tract atrophy. These changes can also affect the quality of life and one’s self-esteem. Hormone replacement therapy (HRT) is considered the best option to achieve therapeutic relief of different menopausal symptoms but is usually restricted to moderate or severe symptoms. Moreover, many women refuse HRT for a variety of reasons concerning the fear of cancer and other adverse effects. According to these considerations, new topics are emerging: Dissatisfaction with drug costs and conventional healthcare, desire for personalized medicines, and the public perception that “natural is good”. In this context, nonhormonal therapies are mostly evolving, and it is not unusual that women often request a “natural” approach for their symptoms. The aim of this study is to investigate nonhormonal therapies that have been identified to reduce the menopausal symptoms.

## 1. Introduction

The onset of menopause is one of the most critical phases in a woman’s life span and is defined retrospectively as the time of the final menstrual period, followed by 12 months of amenorrhea [1,2]. The age at menopause appears to be genetically determined and is unaffected by race, socioeconomic status, age at menarche, or the number of prior ovulations [3]. Menopausal transition, or ‘perimenopause’, is a defined period that begins with the onset of irregular menstrual cycles until the last menstrual period and is followed by fluctuations in reproductive hormones [4,5]. This period is characterized by menstrual irregularities, and prolonged and heavy menstruation intermixed with episodes of amenorrhea, vasomotor symptoms, insomnia, mood issues, and vaginal dryness [6,7]. Hormone replacement therapy (HRT) represents the first choice in the treatment of menopausal symptoms [8,9,10,11]. Moreover, perimenopause can be characterized by unknown fears and ailments: Women can lose their confidence and self-esteem can get shattered with the fast-establishing menopause [7,12]. More than 70 million women in the USA are affected by menopausal symptoms [1,2,13]. Osteoporosis and cardiovascular disease represent the most important long-term effects and seriously impact the quality of life of menopausal women [14,15]. However, nonhormonal therapies are mostly developing and it is not unusual that women often request a “natural” approach for their menopausal symptoms. Nutraceuticals, a pharmaceutical alternative with medicinal properties, extracted from food or plants, belong to this approach [2,16,17,18].

## 2. Treatment Approaches

**Figure 1:**
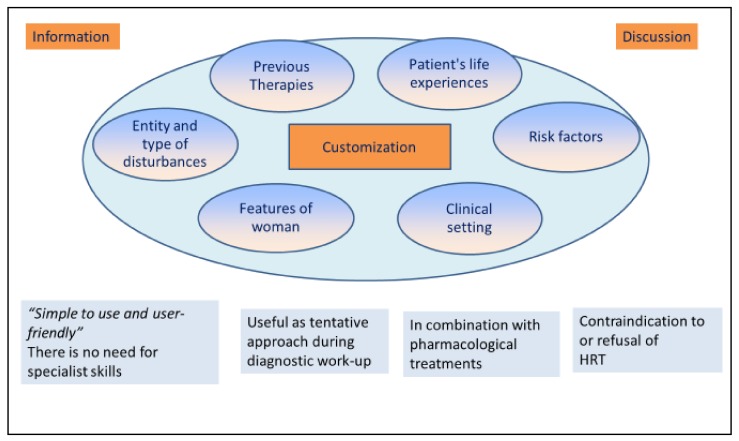
Nutraceutical and the choice of menopausal therapy. Nonhormonal therapies represent a developing option that is characterized by medical information and discussion with the patient. The customization of the therapy is a fundamental point and depends on many factors, clinical and not, such as previous therapies, risk factors, and type of symptoms. Points of strength in nutraceutical choice are: They are user-friendly, are useful as a first approach to menopausal complaints, can be used together with drugs, and are useful if HRT is refused or contraindicated.

Hormone replacement therapy (HRT) is considered the best option to achieve therapeutic relief of different menopausal symptoms [10,19]. U.S. Food and Drug Administration (FDA) indications include HRT for vasomotor symptoms, for prevention of bone loss, for the genitourinary syndrome of menopause, and for premature hypoestrogenism [10,20,21,22,23]. Appropriate treatment includes an early administration of HRT before the age of 60 and up to ten years of amenorrhea [10,11,13,24]. Customization of the dose, routes of administration, types of combination, annual controls, and a treatment duration less than five years are guarantees of a good risk/benefit ratio [10]. The estrogens prescribed are mainly ethinyl estradiol, conjugated equine estrogens (CEE), synthetic conjugated estrogens, and micronized 17b-estradiol [10,13]. Progestogen is used to prevent endometrial thickening and the increase of risk of endometrial cancer during estrogen therapy [10,25]. Progestins more often prescribed are medroxyprogesterone acetate (MPA), norethindrone acetate, and native progesterone [10,26]. Bazedoxifene is a new compound that belongs to the selective estrogen receptor modulators (SERM) and is used with CEE to improve the tissue selectivity [27,28]. However, the appropriate dose of HRT should be determined individually in order to reduce adverse effects, such as fluid retention, nausea, headaches, breast tenderness, bloating, leg cramps, and vaginal bleeding [10,11,14,15,29,30]. Nevertheless, HRT use is usually restricted to moderate or severe symptoms [10,19,31]. Absolute contraindications are represented by undiagnosed abnormal vaginal bleeding, active thromboembolic disorder or acute-phase myocardial infarction, suspected or active breast or endometrial cancer, and active liver disease with abnormal liver function tests [19,32]. Endometriosis is considered as a relative contraindication; in fact, the absolute risk of disease recurrence and malignant transformation is unknown, and the impact of HRT use on these outcomes is difficult to quantify [33,34,35]. Even if it is well-known that HRT is the gold standard treatment for symptomatic menopausal women, it has been reported that less than 30% of menopausal women take HRT and only 15% continue the therapy for a prolonged period [36,37,38]. Twenty-five percent of women remain symptomatic for more than five years, and almost 15% of the 60s and 9% of the 70s have significant vasomotor symptoms; in these kinds of patients, there is no agreement to use HRT [39]. Moreover, many women refuse HRT for a variety of reasons concerning the fear of cancer and adverse effects such as weight gain [40]. Alongside these considerations, new concepts are emerging: Consumers’ dissatisfaction with drug costs and conventional healthcare, desire for personalized medicines, the turn to natural products for treatment and prevention, a new focus on preventive medicine, and the public perception that “natural is good” [2,16,37,41,42,43], as reported in Figure 1.

In such a scenario, nonhormonal therapies are mostly developing, and it is not unusual that women often request a “natural” approach for their symptoms. Apart from much personal skepticism, the ability to listen and to grasp the needs of patients is particularly relevant in menopausal women who are going through this critical phase of life [3,44]. This aspect, which has so far been underestimated, has recently been investigated, highlighting the needs of women about the topic: Women want their healthcare providers to start listening to what they report, want to discuss and seek help for nonvasomotor menopause-related symptoms, and want clear evidence-based information about the various hormonal and nonhormonal treatment options [40,44]. In the last years, nutraceuticals have gained immense popularity when compared with HRT due to their claimed ability to relieve menopausal symptoms [45,46]. Nutraceuticals are foods, parts of foods, and botanicals that provide medical or health benefits, including the prevention and treatment of disease [47,48]. The term “nutraceutical” comes from two words: “nutrient” (food component) and “pharmaceutical” (medical drug) and the name was coined in the last century by Stephen De Felice, founder and chairman of the Foundation for Innovation in Medicine, an American nonprofit organization [49]. The philosophy behind nutraceuticals was probably introduced in Asia throughout ancient China and then improved and defined by physicians of Kampo medicine, the study of traditional Chinese medicine in Japan, especially since the seventh century [50]. Kampo has a holistic therapeutic approach, as it considers the mind and body like one entity: The therapeutic aim is to alleviate symptoms and to bring back harmony in bodily functions [51,52,53]. However, the traditional Chinese medicine (TCM) includes several therapeutic approaches, such as acupuncture and moxibustion, for menopausal complaints [54,55,56]. Moreover, in TCM, menopause is considered as a kidney dysfunction [55,56]. This organ is firstly conceptualized as responsible for fluid balance, temperature, and fertility; and secondly, impacts the function of the heart, spleen, and liver, the latter being considered as the center of emotions [55,56]. For the determination of the appropriate herbal prescription, the physician investigates the complaints and symptoms of the patient, including taking their temperature, examining sensation, weakness, or sweating, symptoms which are not often primarily taken into account in conventional medicine [50,57]. To date nutraceuticals include: Dietary supplements (substances which have established nutritional functions able to affect structure and function of body such as vitamins, minerals, amino acids, fatty acids, probiotics, prebiotics, antioxidants, enzymes, coenzyme Q, carnitine, etc.), herbal medicines (isoflavones, pollen extracts, cimicifuga, red clover, etc.), functional foods—any modified food or ingredient that may provide a benefit (prebiotics-oligofructose, omega-3, canola oil, stanols), and medicinal foods (transgenic cows and lactoferrin for immune enhancement, transgenic plants for oral vaccination against infectious diseases, health bars with added medications). Among these, herbal medicines including isoflavones, black cohosh, red clover, pollen extracts, and others may be used in symptomatic menopausal women. In the US and Britain, surveys show that 80% of peri- and postmenopausal women are current or former users of dietary supplements [58]. However, the benefits of these compound have yet to be demonstrated with certainty, and these regimens are not completely free from side effects [8,37,59,60,61]. Nutraceuticals are considered differently depending on a country’s legislation. In the European community, they are placed in the middle ground between drugs and food: They are extracted from food or plants with medicinal properties [18,47]. In this scenario, nutraceuticals have a role in the management of symptomatic menopausal women.

## 3. Phytoestrogens

Phytoestrogens are presently the most popular form of alternative therapy for support of menopausal symptoms, besides HRT [62,63]. They are plant-based compounds in about 300 plants [35]. Its name comes from the Greek word phyto (“plant”) and estrogen. The main classes are isoflavones (active in humans), lignans (active in humans), cumestan, and lactones [64]. Food sources are various: soy flour, legumes, fruits and vegetables, cereals, olive oil, wheat, etc. Their chemical structure and efficacy are almost similar to oestradiol [63].

### Isoflavones

Isoflavones are the most important compound of phytoestrogens and are produced almost exclusively by the members of the Fabaceae like bean. It includes daidzein, genistein, biochanin A, formononetin, and glycitein [63]. They showed agonist–antagonist estrogen action and exerted elective stimulation of β-estrogen receptors (βERs) with less affinity and lower potency than estrogens [64]. Moreover, they stimulate the synthesis of sex hormone binding globulin (SHBG); therefore, safety in long-term use could be expected [63]. The examination of meta-analyses of randomized controlled trials to evaluate the effectiveness of phytoestrogens in vasomotor symptoms and their side effects in postmenopausal women revealed considerable divergence among authors [63]. Nevertheless, most reported mitigation of the symptoms, as well as improvement in the quality of life; none reported any side effects. Another recent review argued that no conclusive evidence showed a benefit of phytoestrogen-enriched or -derived products for menopausal vasomotor symptoms, except for products containing a minimum of 30 mg per day of genistein [64]. It is well known that the absorption of the soy isoflavones depends on the presence of the intestinal flora that are capable of producing glycosidases and therefore to hydrolyze genistein and daidzin to the active aglycons [65,66]. Taking this into consideration, it has been suggested to combine soy isoflavones with lactic acid bacteria in the form of spores, resistant to the gastric and biliary secretion, to assure the bioavailability of soy isoflavones [67,68]. The association with probiotic was also studied for symptoms of genitourinary syndrome of menopause, but results were not satisfactory [69]. Isoflavones exert a limited beneficial effect on cognition, as increased choline acetyltransferase and brain-derived neurotrophic factor in the hippocampus and frontal cortex [70]. However, this effect may be modified by age, gender, ethnicity, menopausal status, and length of treatment [70]. The effects on bone metabolism are interesting due to a significant decrease in bone resorption process, especially if associated with HRT [26]. Moreover, the topical application showed a good effect on vaginal health and dyspareunia. Finally, several studies showed a significant effect on the lipid profile and inflammatory marker associated, with a lower risk of cardiovascular disease [71,72].

## 4. Herbal Derivatives

Herbal remedies are frequently used to alleviate menopause symptoms and are effective in the treatment of acute menopausal syndrome with different mechanisms [73]. One of the major problems is that people usually take herbal therapies in the form of supplement pills and not as a preparation made directly from the herb by a trained herbalist [74]. Moreover, herbal supplements are not as strictly regulated as prescription drugs and quality, safety, and purity may vary between brands or even between bundles of the same brand [75]. These compounds may also interact with prescription drugs, resulting in dangerous changes in the effect of the drug [74,75]. Here below a list of the herbs most frequently used in the treatment of menopausal symptoms, also reported in Table 1.

### 4.1. Actaea racemosa

This herb, also called *Cimicifuga racemosa* or black cohosh or fairy candle, has a long history of use: Native Americans used it to treat many diseases like musculoskeletal pain, fever, cough, pneumonia, sluggish labor, and menstrual irregularities [76]. It’s among the most studied herbal derivatives with observational studies during the 50s and 70s, and controlled studies since the 80s for a total of 11,073 patients. Black cohosh showed a positive effect in treating hot flashes and other menopausal symptoms like sleep quality. According to a recent study, the herb can also inhibit the growth of the myomas, in contrast to tibolone in patients with uterine myomas [77]. However, evidence on the safety of black cohosh was inconclusive, owing to poor reporting. A review argued that there is insufficient evidence to support the use of black cohosh for menopausal symptoms, particularly concerning allocation concealment and the handling of incomplete data from studies [78]. When looking at recent data, evidence of effectiveness for black cohosh has improved, with good evidence existing for standardized isopropanolic extract preparations of this herb, such as those approved for use in treatment in many European countries [76]. Terpene glycosides are the active compounds and bind to the estrogen receptor and selectively suppress the secretion of LH without any effect on FSH. Gastrointestinal side effects are the most common and there has been some concern about hepatotoxicity with long-term use of black cohosh [76,77].

### 4.2. Evening Primrose Oil

Also called *Oenothera biennis* oil, it contains omega-6 fatty acids, which increase prostaglandin E2 that has anti-inflammatory effects [79]. The main application is for systemic diseases marked by chronic inflammation, such as atopic dermatitis and rheumatoid arthritis [80]. It is often used for several complaints such as menopausal and premenstrual symptoms [79]. However, several studies showed that the compound has no benefit in treating menopausal flushing compared with placebo [80]. *Oenothera biennis* oil may cause mild gastrointestinal side effects or lower seizure threshold in patients taking antiepileptic drugs [79,80].

### 4.3. Foeniculum vulgare

The common name of this herb is Fennel. It is characterized by the presence of palmitic acid and beta-sitosterol and shows antiandrogenic and anti-inflammatory effects [81,82]. Its main application is on hot flashes in postmenopausal women but it can also help anxiety in those patients with depression [81]. Vaginal fennel ethanol extract cream showed an improvement of vaginal atrophy and sexual functions in menopause women due to its estrogenic effects [83]. No critical side effects have been reported [81,83].

### 4.4. Ginkgo biloba

The herb was used in the treatment of attention disorders in postmenopausal women, but several studies reduced its positive action [84]. However, a recent study showed a positive effect on the sexual desire of menopausal women [85]. The side effects include mild gastrointestinal disorders, allergic reactions, headache, and lowering of seizure threshold [84,85].

### 4.5. Glycyrrhiza glabra

It is more famously called licorice and contains terpenes, saponins, flavonoids, isoflavonoids, and steroids [86]. It has various levels of estrogenic activities, and one clinical trial study showed that it is more effective than HRT in improving hot flash duration [86]. However, HRT can reduce the duration and severity of hot flashes more than licorice [86]. Prolonged use of this herb can cause cardiovascular disease, hypercortisolism, hypokalemia, and hypernatremia and safety studies are necessary [87].

### 4.6. Hypericum perforatum

The herb, also called St. John’s Wort, showed a positive effect on the treatment for the vasomotor symptoms of postmenopausal women [88]. A study found a chemopreventive effect in human breast cancer cells through AMPK/mTOR signaling [89]. A systematic review showed that the combination of this compound with *C. racemosa* demonstrated a positive effect on climacteric complaints [90]. The side effects are fewer and include gastrointestinal discomfort, sensitivity to light, restlessness, and fatigue [91].

### 4.7. Medicago sativa

Also called Alfalfa, this herb contains noncellulosic polysaccharides that exert various effects: Immunomodulatory, anti-inflammatory, antioxidant/anticancer, and growth-promoting bioactivities and, in addition, it seems to reduce the incidence of chronic disease [92]. It also shows a slight effect on neurovegetative menopausal symptoms [93]. Some critical side effect besides the infection were salmonella, escherichia coli, and listeria, which reduced its use [94,95].

### 4.8. Melissa officinalis

This herb, also known as lemon balm, bee balm, or honey balm, has long been used as a medicinal plant but also as a vegetable and to add flavor to dishes [96]. It contains volatile compounds, triterpenoids, phenolic acids, and flavonoids [97]. It has been used for the treatment of a wide range of diseases, especially anxiety and some other mental disorders [98]. It shows many pharmacological effects, such as anxiolytic, antiviral, antioxidant, and antispasmodic activities but also exerts action on the central nervous system, mainly on cognition and memory [99,100]. One of its derivatives is caffeic acid, and no dangerous side effects are reported in the literature; however, confirmatory trials are warranted to substantiate these effects in the clinical setting [101].

### 4.9. Panax ginseng

Anti-inflammatory properties characterize this plant, and a recent review of randomized clinical trials showed promising results for improving glucose metabolism and moderating the immune response [102]. Possible mechanisms of action of ginseng include hormonal effects related to those of estrogen with a slight effect on depression, mood disorders, and sexual function [103]. The principal active compounds are ginsenosides, which have been shown to exert estrogen-like actions [104,105]. However, side effects are not clear at all, and more studies are requested to assess the effects on hot flash frequency and endometrial thickness [106].

### 4.10. Passiflora incarnata

Also called passion fruit, it has long been used in traditional herbal medicine for the treatment of insomnia and anxiety, but also a sedative tea in North America [107]. This plant showed analgesic, anti-spasmodic, anti-asthmatic, wormicidal, and sedative actions, but it is also used for dysmenorrhea [107]. It has been proposed to treat early menopausal symptoms such as insomnia, vasomotor symptoms, depression, anger, or headaches [108]. The plant has a good safety profile, and no particular side effects have been reported in the literature, although more studies are needed to widely assess this aspect [109].

### 4.11. Pimpinella anisum

*Pimpinella anisum*, also known as anise, contains an active compound with both estrogenic and analgesic, antioxidant, antimicrobial, anticonvulsant, and antispastic properties [110,111]. Moreover, anise exerts activity on the gastrointestinal system with an antiulcer action while the aromatic effects have been demonstrated in the palliation of nausea [110,112]. The primary therapeutic target in menopause is against hot flashes [111]. No dangerous side effects have been reported in the literature [113].

### 4.12. Salvia officinalis

Also called sage herb, the mechanism of action is exerted through modulation of GABA receptors and serotonin transporters, which impacts on hot flashes and sweats [114]. The active compound inhibits choline esterase in vitro, explaining why excessive use may cause a feeling of warmth, tachycardia dizziness, and epilepsy-like seizures [115]. However, the impact on diabetes and blood pressure drugs are still not clear [116].

### 4.13. Trifolium pretense

Also known as red clover, the oral intake of supplements containing isoflavones of this plant has been reported to be effective in reducing the frequency and severity of hot flashes [117]. Moreover, it shows a chondroprotective effect on inflammation and may be used for preventing osteoporosis [118]. However, this herb is contraindicated with the concomitant use of hormonal drugs [119]. No apparent evidence of adverse events has been shown during short-term use, but there are not enough data on the safety of long-term administration [120].

### 4.14. Trigonella foenum

This herb, also called fenugreek, has been used to treat hot flashes, with some preliminary evidence for prevention of menopausal induced osteopenia [121]. Moreover, some studies have focused on its action for diabetes and dysthyroidism [122]. It contains compounds of mucilage, proteins, and steroidal saponins [121]. No particular dangerous side effects have been reported [121,122].

### 4.15. Valerian officinalis

It is a traditional herb used for the treatment of anxiety and sleep disorders. It showed a sedative effect, probably due to the increase of GABA in the synaptic cleft due to inhibition of its reuptake [123]. It has direct inhibitory effects on the contractility of the human uterus, justifying the traditional use in the treatment of uterine contractions associated with dysmenorrhea [124]. Moreover, this herb is used in the treatment of hot flashes in menopause [125]. No critical side effects have been reported [124,125].

### 4.16. Vitex agnus-castus

*Vitex agnus-castus* (also called chaste tree, chasteberry, or monk’s pepper) increases melatonin release, interacts with opioid receptors, and can play a role in vasomotor symptoms and sleep diseases [126]. It has been used for dysmenorrhea, premenstrual dysphoric disorder, infertility, acne, cyclic breast pain, and diarrhea and flatulence [127]. A recent study showed that *V. agnus-castus* and magnolia, combined with Soy isoflavones + lactobacilli, improve quality of sleep in symptomatic women [128].

## 5. Vitamins

The beneficial effect of vitamins for the treatment of perimenopausal symptoms is limited in the literature [129,130]. Vitamin E could play a decisive role in the prevention of hot flushes if consumed in the amount of 800 IU/day [130]. The protective effect of vitamins E on sleep quality has been recently shown [131]. It could also be an alternative to vaginal estrogen in relieving the symptoms of vaginal atrophy in postmenopausal women [132,133,134]. In postmenopausal women with vitamin D deficiency, isolated supplementation of vitamin D3 were associated with a reduction in the metabolic syndrome risk profile, but also with a lower risk of hypertriglyceridemia and hyperglycemia [135,136,137]. Recent studies focused on other micronutrients such as essential fatty acid, B vitamins, vitamin C, magnesium, and zinc to reducing stress and anxiety [138,139].

## 6. Other Compounds

Recent evidence suggested the role of other sources such as polyphenols extracted from hop or grape seed or lipoproteins of marine origin [140,141,142,143]. These compounds showed a positive role in the relief of menopausal symptoms, especially for vasomotor ones but also other positive effects [140,142,144,145].

## 7. Discussion

HRT represents the first therapeutic option for menopausal symptoms, and its use is supported by a large body of evidence and recommendations of scientific organizations [10,62]. On the contrary, clinical trials about nutraceuticals suffers from some limitations: Uncommon and poorly comparable results, a clear qualitative difference between the available products, a difficult definition of active ingredients, a variable absorption, a different metabolization with the variable presence of active principles, all leading to very variable clinical effects [17,37,146,147,148,149]. Moreover, it needs to be underlined that placebo in all studies can reduce vasomotor symptomatology in between 20% and 50% of women by reducing the intensity of symptoms by more than 30% [64,72]. To be considered effective against vasomotor symptoms, therapies must overcome these “placebo-effects”. Otherwise, we can say that they work as a costly placebo, for which harm cannot be guaranteed without proper studies. Therefore, there is a need for further extensive studies on nutraceutical used for menopausal symptoms. To date, it is a good practice to choose products from factories with good manufacturing practices as for conventional drugs, guaranteeing a consistent, standardized composition and clinical studies to support effectiveness and safety.

## 8. Conclusions

Health care providers should include in discussion with their patients all the available approaches for relief of menopausal symptoms, giving all the information useful for a conscious and shared choice, for real customization of the approach to the complaints in this critical phase of a woman’s life. In this perspective, the nutraceuticals have their strengths because they are “simple to use and user-friendly” with no need for specialist skills, and they represent a concrete choice for women who cannot take HRT [36,38,40,150,151,152,153]. However, managing menopausal disturbances with nutraceutical remedies requires an evidence-based approach. In particular and limited contexts, as indicated for symptomatic women, nutraceuticals are useful as a tentative approach during diagnostic work-up until the final prescription of HRT, for contraindications to or refusal of HRT, in combination with pharmacological treatments.

## Figures and Tables

**Table 1:** Main characteristic of herbal derivatives used to alleviate menopause symptoms.

Herbal Derivatives				
Scientific Name	Common Name	Effects	Side Effects	References
*Actaea racemosa*	Black cohosh	Treatment of menopause symptoms such as hot flash, insomnia, irritability, but also musculoskeletal pain, fever, cough.	Gastrointestinal discomfort.	[76,77]
*Evening Primrose Oil*	Oenothera biennis oil	Treatment for menopausal and premenstrual symptoms, but also for atopic dermatitis and rheumatoid arthritis.	Gastrointestinal disorders and interaction with antiepilectic drugs.	[79,80]
*Foeniculum vulgare*	Fennel	Treatment of hot flashes, anxiety, and vaginal atrophy.	No side effects reported.	[81,82,83]
*Ginkgo biloba*	Ginkgo	Treatment of attention disorders in postmenopausal women.	Gastrointestinal disorders, allergic reactions, headache, and lowering of seizure threshold.	[84,85]
*Glycyrrhiza glabra*	Licorice	Treatment of hot flash duration.	Cardiovascular disease, hypercortisolism, hypokalemia, and hypernatremia.	[86,87]
*Hypericum perforatum*	St. John’s Wort	Treatment for the vasomotor symptoms of postmenopausal women.	Gastrointestinal disease, sensitivity to light, fatigue.	[88,89,90,91]
*Medicago sativa*	Alfalfa	Effect on neurovegetative menopausal symptoms.	Possible infection with Salmonella, Escherichia coli, and Listeria.	[92,93,94,95]
*Melissa officinalis*	Lemon balm, bee balm or honey balm	Effect on anxiety.	No side effect reported.	[96,98,99,100,101]
*Panax ginseng*	Ginseng	Treatment of sleep disorders, depression, and sexual function.	Possible effect on endometrial thickness.	[102,103,104,105,106]
*Passiflora incarnata*	Passion fruit	Treatment of vasomotor symptoms, insomnia, anxiety and dysmenorrhea.	No side effect reported.	[107,108,109]
*Pimpinella anisum*	Anise	Treatment of hot flashes but it also exerts an antiulcer action.	No side effects reported.	[110,111,112,113]
*Salvia officinalis*	Sage herb	Treatment of hot flashes and sweats.	Possible interaction with diabetes and blood pressure.	[114,115,116]
*Trifolium pretense*	Red clover	Treatment of hot flashes and it also exerts a bone preventing loss.	No side effects reported.	[117,118,119,120]
*Trigonella foenum*	Fenugreek	Treatment for hot flashes and osteopenia.	No particularly side effects.	[121,122]
*Valerian officinalis*	Valerian	Useful for hot flashes, anxiety, sleep disorders and dysmenorrhea.	No side effects reported.	[123,124,125]
*Vitex agnus-castus*	Chaste tree, chasteberry or monk’s pepper	Treatment for vasomotor symptoms and sleep diseases.	Not reported.	[126,127,128]
